# Experimental and Spectral Analysis of the Wake Velocity Effect in a 3D Falcon Prototype with Oscillating Feathers and Its Application in HAWT with Biomimetic Vortex Generators Using CFD

**DOI:** 10.3390/biomimetics10090622

**Published:** 2025-09-16

**Authors:** Hector G. Parra, Javier A. Guacaneme, Elvis E. Gaona, Hernán Dario Cerón-Muñoz

**Affiliations:** 1 Engineering Faculty Cajicá, Universidad Militar Nueva Granada, Bogotá 111321, Colombia; 2 Facultad de Ingeniería, Universidad Distrital Francisco José de Caldas, Bogotá 110231, Colombia; jguacaneme@udistrital.edu.co (J.A.G.); egaona@udistrital.edu.co (E.E.G.); 3 Escola de Engenharia de São Carlos, Universidade de São Paulo, São Carlos 13566-590, Brazil; hernan@sc.usp.br

**Keywords:** peregrine falcon, spectrum, vortex generators, HAWT, CFD simulations

## Abstract

The peregrine falcon, known as the fastest bird in the world, has been studied for its ability to stabilize during high-speed dives, a capability attributed to the configuration of its dorsal feathers. These feathers have inspired the design of vortex generators devices that promote controlled turbulence to delay boundary layer separation on aircraft wings and turbine blades. This study presents an experimental wind tunnel investigation of a bio-inspired peregrine falcon prototype, equipped with movable artificial feathers, a hot-wire anemometer, and a 3D accelerometer. Wake velocity profiles measured behind the prototype revealed fluctuations associated with feather motion. Spectral analysis of the velocity signals, recorded with oscillating feathers at a wind tunnel speed of 10 m/s, showed attenuation of specific frequency components, suggesting that feather dynamics may help mitigate wake fluctuations induced by structural vibrations. Three-dimensional acceleration measurements indicated that prototype vibrations remained below 1 g, with peak differences along the X and Z axes ranging from −0.06 g to 0.06 g, demonstrating the sensitivity of the vibration sensing system. Root Mean Square (RMS) values of velocity signals increased with wind tunnel speed but decreased as the feather inclination angle rose. When the mean value was subtracted from the signal, higher RMS variability was observed, reflecting increased flow disturbance from feather movement. Fast Fourier Transform (FFT) analysis revealed that, for fixed feather angles, spectral magnitudes increased uniformly with wind speed. In contrast, dynamic feather oscillation produced distinctive frequency peaks, highlighting the feather’s influence on the wake structure in the frequency domain. To complement the experimental findings, 3D CFD simulations were conducted on two HAWT-type wind turbines—one with bio-inspired vortex generators and one without. The simulations showed a significant reduction in turbulent kinetic energy contours in the wake of the modified turbine, particularly in the Y-Z plane, compared to the baseline configuration.

## 1. Introduction

The development of wind energy conversion systems worldwide has become a well-established field of research and technological innovation. Several research efforts focus on the implementation of strategies for structural vibration mitigation in wind turbines [1,2,3,4], while others are directed toward the design and integration of active vortex generator devices [5,6].

Computational Fluid Dynamics (CFD) simulation serves as a fundamental tool to complement the analysis of these mechanical modifications [7,8,9,10]. Recent studies have explored the incorporation of bioinspired solutions, particularly from avian species, as a strategy to optimize aerodynamic performance [11,12,13]. Morphological and functional characteristics developed through evolutionary processes in certain species are used as references to improve the turbulence behavior in aerial systems [14,15,16,17]. Bioinspiration, or biomimetics, seeks to integrate physiological traits found in animal or plant species into human-engineered systems with the aim of enhancing their operational efficiency.

In engineering, such approaches have shown significant progress in the naval and aeronautical sectors. In naval applications, bioinspired strategies aim to improve maneuverability and propulsion efficiency of marine vessels, while in the aeronautical domain, the focus is on enhancing aerodynamic performance and reducing structural noise in aircraft and wind turbines 

This article focuses on understanding and quantifying the effect of the falcon’s feathers and their application through the development of devices aimed at improving the vibrational response of structures such as aircraft and wind turbines.

## 2. Materials and Methods

**Figure 1:**
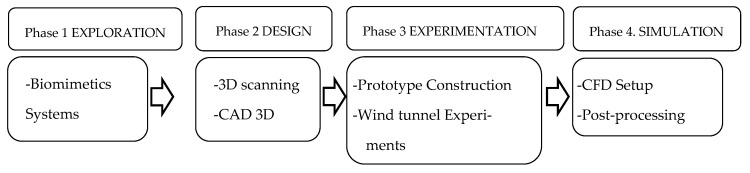
Methodology. The blocks illustrate the sequential progression of activities within each phase.

The methodology developed to analyze the effect of the falcon’s feathers, and their potential applications consists of a series of experiments carried out in four phases, as illustrated in Figure 1.

The 3D accelerometer used has a resolution of 0.001 g and an accuracy of ±0.005 g. The hot-wire anemometer used for wind velocity measurements has a resolution of 0.01 m/s, as specified by the manufacturer. To ensure measurement repeatability, each test condition was repeated three times, and the standard deviation of the readings was analyzed. The results showed consistent behavior across the trials, confirming the reliability of the data acquisition system generators based on bioinspired principles [18,19,20], can contribute to reducing noise generated by turbines and increasing energy output [21].

**Figure 2:**
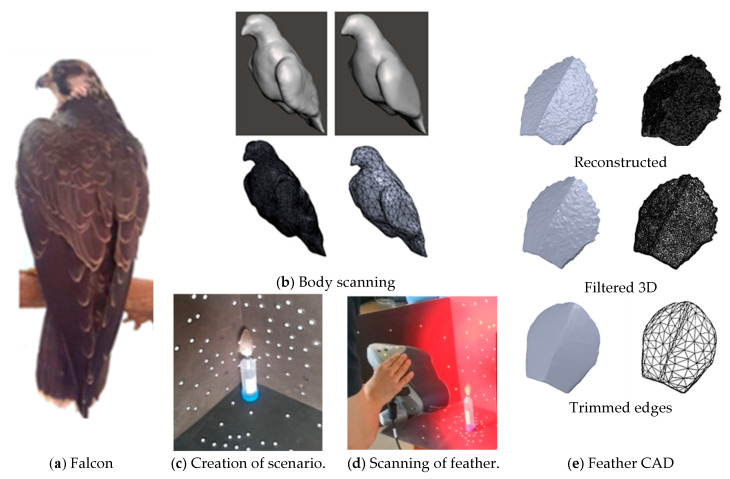
Scanning process.

The peregrine falcon (Figure 2) is a bird capable of reaching diving speeds close to 350 km/h, making it the fastest aerial predator in the world [9]. Figure 2 shows that, using 3D scanning and a dissected specimen, a CAD 3D model of the bird’s body and feathers was obtained [22]. There is commercially available vortex generators used on the blade surfaces of wind turbines. The design of active vortex generators based on bioinspired principles [23], [24] can contribute to reducing noise generated by wind turbines and increasing energy output [25].

## 3. Results

With the support of the Jaime Duque Zoo in Briceño, Colombia, a 3D scan of a taxidermized peregrine falcon, along with biological samples of its dorsal feathers, was obtained for analysis.

Once the point cloud is obtained, a solid model is generated using MESHLAB V2023.12^®^. The surface roughness of the 3D model is then filtered and smoothed using MESHMIXER^®^, resulting in a refined 3D solid suitable for meshing and subsequent simulation in a CFD environment (Figure 2).

**Figure 3:**
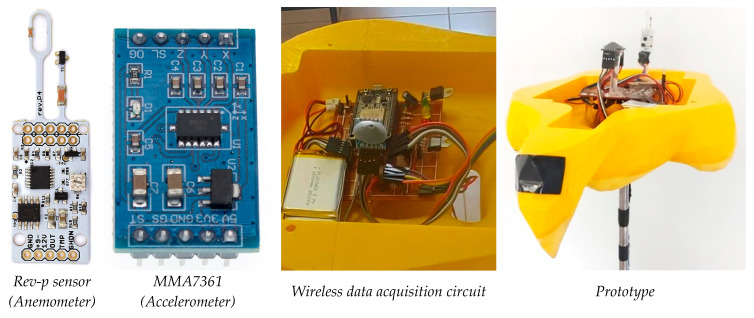
Prototype construction.

Following the additive manufacturing of the falcon prototype, a servomechanism was integrated to allow controlled adjustment of the angular displacement of the dorsal feathers. A “V”-shaped configuration was implemented, replicating the anatomical orientation observed in the biological specimen (indicated by yellow circles), as illustrated in Figure 3.

**Figure 4:**
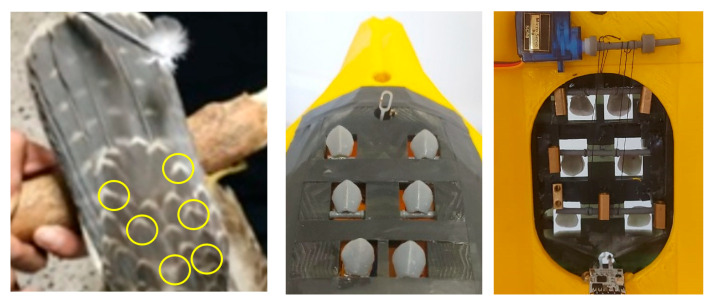
Dorsal feathers in “V” shaped with servo mechanism.

Figure 4 illustrates the design and implementation of a servomechanism that, using control wires, enables the rotation of the feather support shafts, allowing precise adjustment of their angle of inclination.

The selection of the six dorsal feathers for actuation was based on prior morphological and aerodynamic analyses of peregrine falcon wing structures, which indicated that the dorsal region near the wingtip plays a critical role in flow modulation during gliding and maneuvering. These feathers were found to be the most mobile and responsive to dynamic changes in airflow. Additionally, their position near the trailing edge provides a favorable location for influencing boundary layer behavior and wake dynamics. This region was thus chosen to replicate the bioinspired vortex generation mechanism with the greatest potential impact on aerodynamic performance and structural vibration attenuation.

This mechanism is interfaced with an embedded system equipped with Bluetooth connectivity, facilitating integration with a software application developed in MATLAB V2023.12^®^. Clearance gaps between the feathers and the bird’s body were intentionally incorporated to allow unrestricted feather articulation and to achieve a zero-degree inclination position.

The tunnel used is an open-type wind tunnel with a test chamber volume of 0.46 × 0.46 × 1.2 m^3^. It is made of fiberglass and has a wind speed range from 0 to 25 m/s. It includes a conical diffuser and a three-phase fan controlled by a variable frequency drive. The wind tunnel is located at the School of Engineering (EESC) of the University of São Paulo (USP), São Carlos, Brazil.

**Figure 5:**
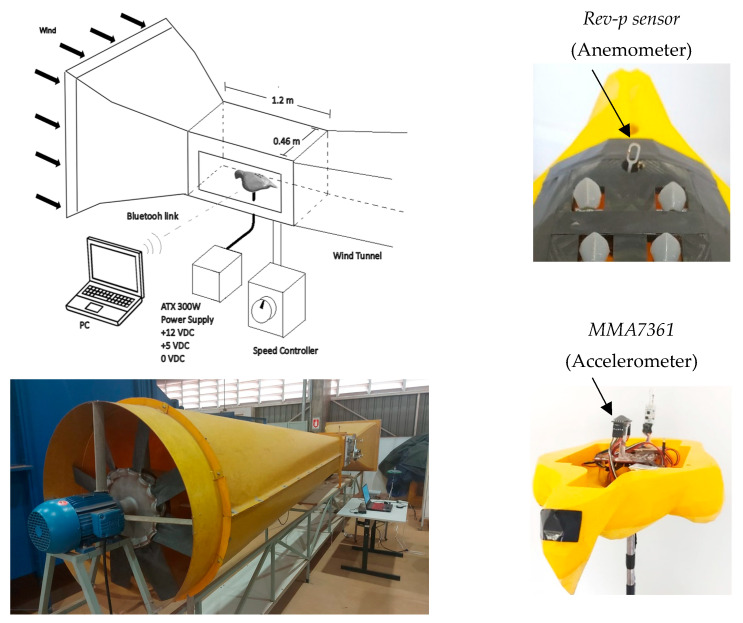
Experimental setup.

Figure 5 presents the experimental setup of the wind tunnel test designed to analyze the aerodynamic behavior of the 3D falcon model. The prototype is equipped with an electronic circuit and wireless sensors, and due to the specific power requirements of the hot-wire sensor (Rev-p sensor), it was necessary to employ a DC ATX power supply unit providing +12 VDC, +5 VDC, and 0 VDC outputs.

The 3D accelerometer used has a resolution of 0.001 g and an accuracy of ±0.005 g. The hot-wire anemometer used for wind velocity measurements has a resolution of 0.01 m/s, as specified by the manufacturer. To ensure measurement repeatability, each test condition was repeated three times, and the standard deviation of the readings was analyzed. The results showed consistent behavior across the trials, confirming the reliability of the data acquisition system.

**Figure 6:**
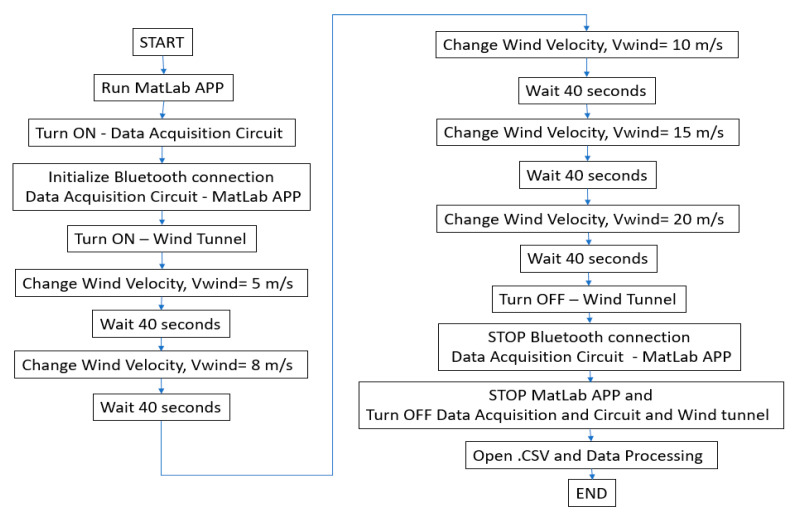
Wind tunnel measurement protocol.

Based on the operation of the available subsonic wind tunnel and technical recommendations from expert personnel (Figure 6), a measurement procedure was established to analyze changes in the wake flow generated by the peregrine falcon prototype under varying wind speed conditions. Data acquisition windows of 40 s were defined to ensure a sufficient and manageable dataset for post-processing and analysis.

**Figure 7:**
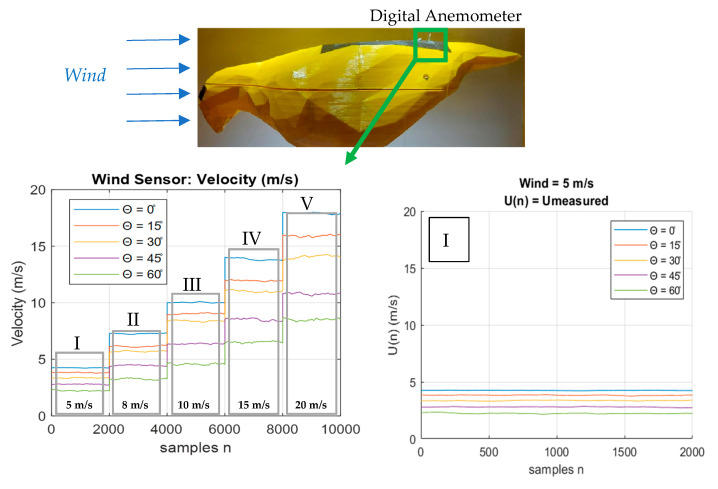

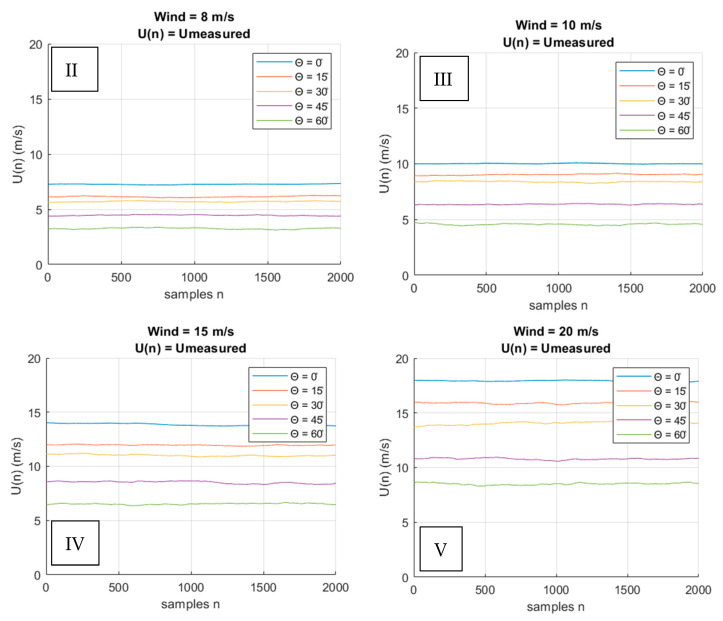
Wind velocity values segmented.

Figure 7 presents the wind velocity measurements obtained using a digital anemometer for various feather inclination angles. At a tunnel wind speed of 5 m/s, a progressive decrease in the measured velocity is observed as the feather angle increases. The wind velocity remains approximately constant throughout the sampling period.

The third section of Figure 7 corresponds to a tunnel wind velocity of *Vwind* = 10 m/s. In this plot, greater separation between the velocity curves associated with different feather angles becomes evident, indicating a more pronounced reduction in wake velocity generated by the feathers.

At a tunnel wind speed of *Vwind* = 15 m/s, the velocity measured by the sensor positioned 20 mm downstream from the feathers exhibits increased fluctuations compared to measurements at lower wind speeds. For *Vwind* = 20 m/s, both the separation between velocity curves and the amplitude of fluctuations become more significant relative to the signals measured at lower wind speeds (Figure 7).

**Figure 8:**
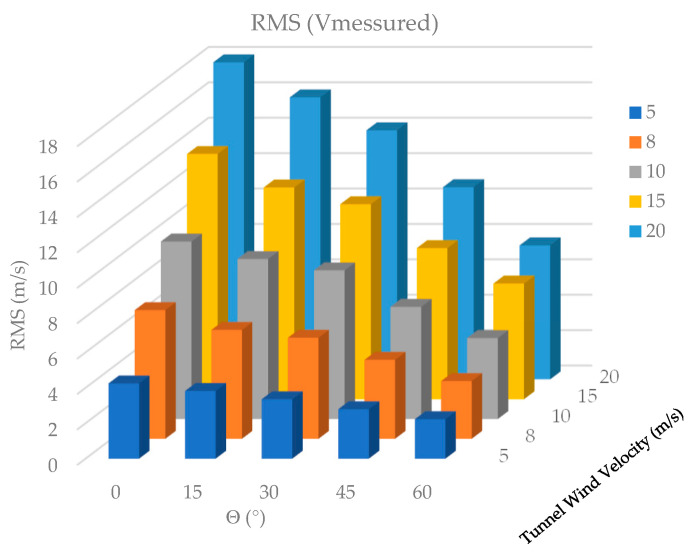
RMS values.

Due to the consistent pattern observed in the shape of the velocity curves across different wind speeds, a comparative plot of the Root Mean Square (RMS) values was generated, as shown in Figure 8.

Figure 8 shows that the RMS value of the wind velocity measured behind the feathers increases as the wind velocity in the tunnel increases and decreases as the pitch angle of the prototype feathers increases. To better observe the fluctuations of the velocity, present due to the variation in the feather tilt angle, the average value of each signal is subtracted and plotted.

**Figure 9:**
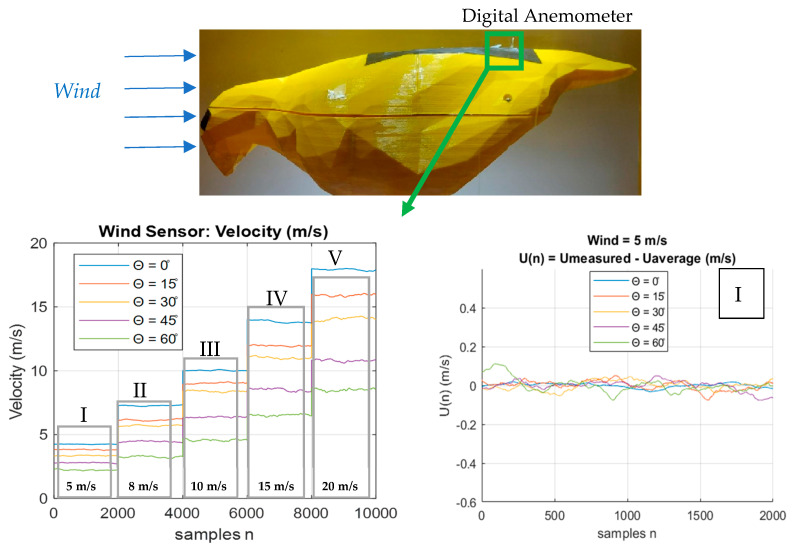

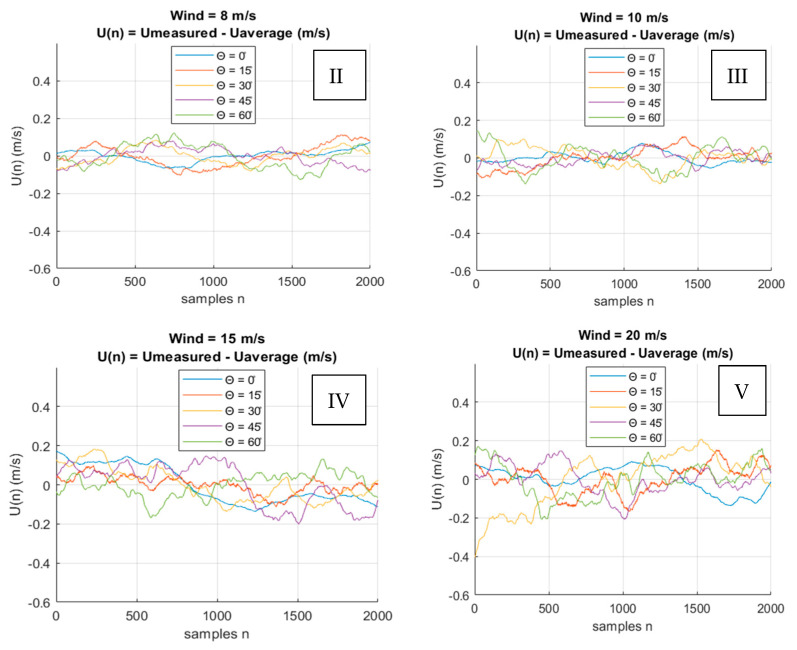
Wind velocity segment without average value.

Figure 9 shows the signals measured without average value. For a tunnel velocity of 8 m/s, the figure shows a larger increase in velocity fluctuations than that observed at 5 m/s. A sinusoidal behavior composed of low-frequency and high-frequency signals with low amplitude, associated with the behavior of the particles in the wind wake generated by the feathers, is also observed.

**Figure 10:**
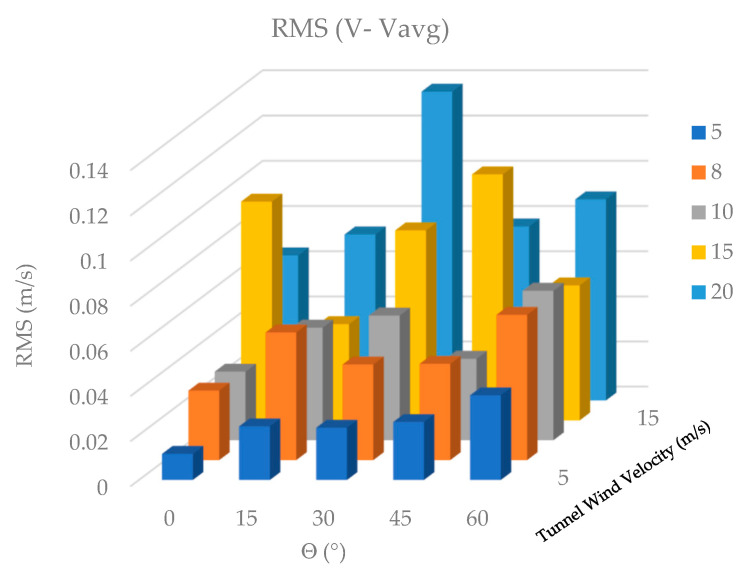
RMS (U-Average) values.

It is difficult to see differences between curves. For this reason, it is proposed to obtain the RMS value and compare (Figure 10). The maximum values of the green and purple curves indicate that increasing the wind velocity in the tunnel increases the amplitudes of wake fluctuations generated by the feathers (Figure 9).

**Figure 11:**
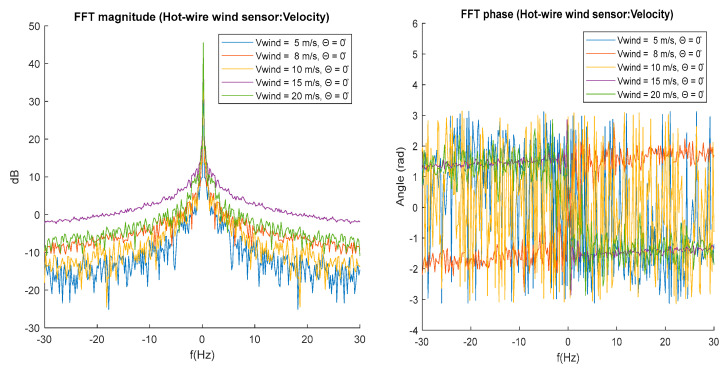
Magnitude and phase spectrums for theta Θ = 0°.

Wind values measured with *Vwind* = 20 m/s present higher amplitudes at theta angles 30 and 45 degrees. Measured velocity fluctuations when *Vwind* = 10 m/s and *Vwind* = 8 m/s are very similar in terms of peak amplitudes and shapes; however, this can only be observed by more detailed spectral analysis (Figure 11).

Figure 10 shows that the RMS value of wind velocity, calculated after removing the mean, increases near the feathers as wind tunnel velocity increases. The RMS values fluctuate in the bar graph as the feather tilt angle changes, primarily due to the subtraction of the mean. To complement the time-domain analysis of the wind velocity signals measured with the hot-wire anemometer, the magnitude and phase of the spectral components were computed using the Fast Fourier Transform (FFT) function in MATLAB V2023.12^®^.

Figure 11 shows the magnitude spectrum for the wind signals measured with a fixed feather tilt angle theta = 0°. The magnitude spectrum shows an increase in the spectral components of the measured wind signals if the wind velocity in the tunnel increases. for frequencies higher than 1 Hz the magnitude spectral curves for 15 and 20 m/s wind wake show smaller fluctuations between spectral components with respect to the 5 and 10 m/s velocities. The phase spectrum in Figure 11 shows the angle associated with each spectral component, the phase is the angle shift in the spectral component. The phase spectrum presents greater variance for the signals measured at wind velocities of 5, 10, and 20 m/s.

**Figure 12:**
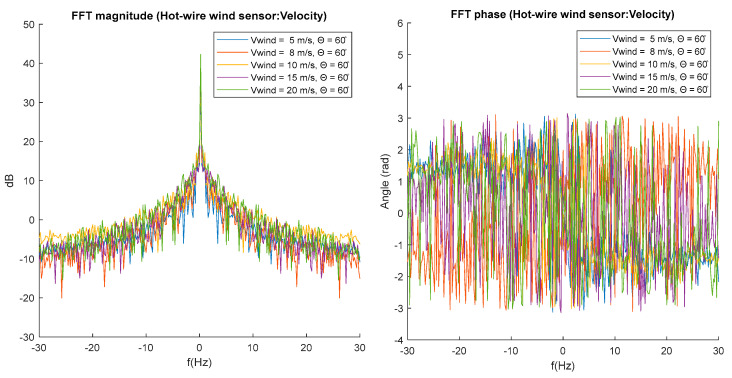
Magnitude and phase spectrums for theta = 60°.

To better analyze the effect generated by the feather tilt, the magnitude and phase spectra are analyzed for theta = 60°. This spectrum shows clustering or similarity of the spectral components of Figure 11, indicating that the feathers at a fixed theta angle of 60° can help reduce several high-frequency components in the wake generated by the bird, but increase several low-frequency components. The phase spectrum (Figure 12) shows very similar behavior between signals measured at different velocities with high variance between their components.

**Figure 13:**
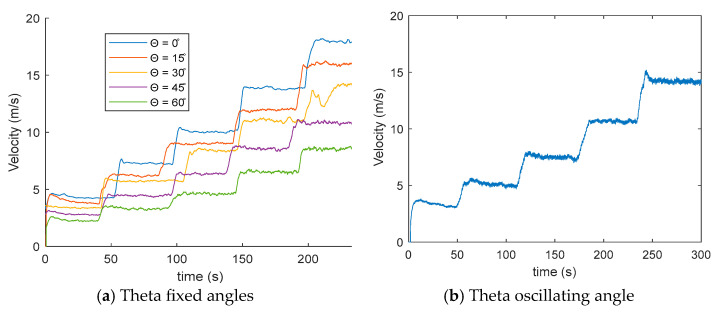
Wind velocity values of digital anemometer sensor for theta fixed angles and theta oscillating angles.

**Figure 14:**
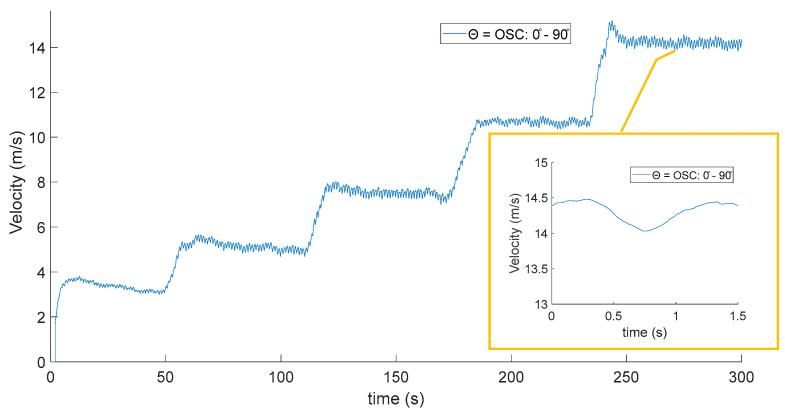
Wind velocity values of digital anemometer for oscillating feathers—Zoom.

Figure 13 shows the wind signal measured by the anemometer with *f_osc_* = 1 Hz, performing wind velocity changes in the tunnel at 5, 8, 10, 15, and 20 m/s; the figure shows in the zoom an oscillatory behavior as the wind velocity in the tunnel stabilizes, so the observed oscillations are associated with fluctuations generated in the wind wake by the mechanic oscillation of the feathers (Figure 14).

Finally, a measurement of the wind wake behavior when the feathers oscillate from 0 to 90 degrees with oscillation frequency *f_osc_* = 1 Hz was carried out using a servomotor. The measured wind velocity curve is plotted as a function for comparison with the measured wind velocity curves measured with the feathers at fixed angles (Figure 13b).

**Figure 15:**
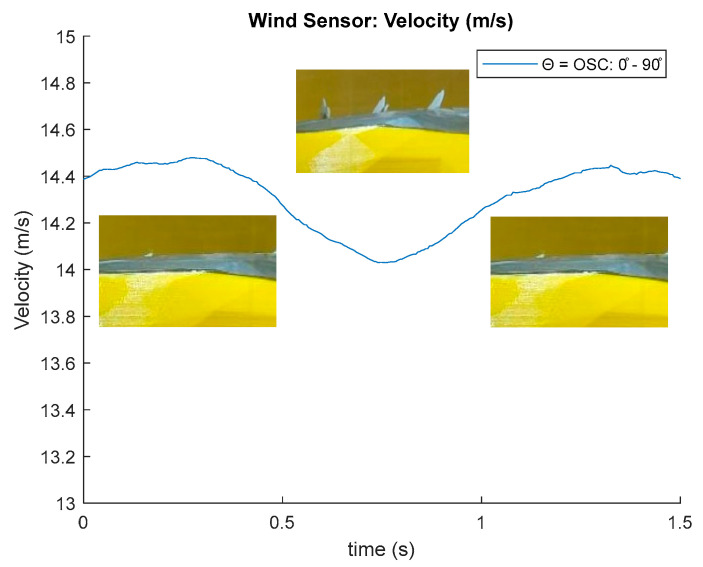
Wind velocity values digital anemometer for oscillating feathers.

**Figure 16:**
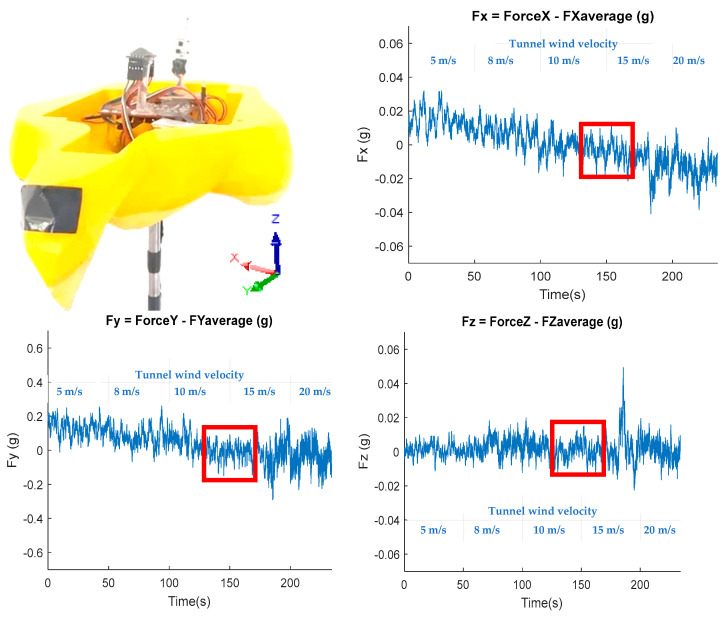
Accelerations 3D without average value for oscillating feathers.

Figure 15 shows the behavior of the accelerations in the center of the falcon prototype with oscillating feathers with *f_osc_* = 1 Hz with no mean value. Figure 16 shows signals associated with structural vibration or acceleration with a high variance and more visible increases in the X-axis associated with the increase in the tunnel wind velocity.

The measured Y-axis vibration shows an increasing behavior associated with increasing tunnel wind velocity from 170 s onwards. The force measured with accelerometer in the Z direction, as well as in the X and Y axes, shows an increase in amplitudes from 170 s onwards. The wind velocity increases the vibrations experienced by the prototype.

In the red boxes, a reduction in the measured acceleration peaks can be observed within a wind tunnel velocity range of approximately 10 to 15 m/s (Figure 15). The magnitude spectrum in Figure 16 shows the amplitudes of the measured wind velocity signal components generated by the oscillating feathers at 20 mm distance.

This spectrum allows the observation of characteristic peak values that are associated with the oscillation frequency, indicating that the acquisition system is sensitive to measure changes in the wake of the prototype, the feather oscillations increase the values of the spectral components in the range: 0–10 Hz.

**Figure 17:**
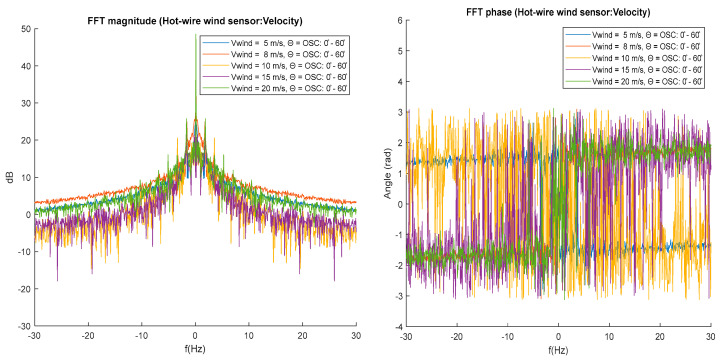
Magnitude and phase spectrums for theta = 60°.

Figure 17 shows the phase spectrum with high variance among its components, although a reduction in variance is observed for the spectra at high velocities, indicating less rapid phase changes with respect to the phase spectra performed at the wind velocity signals measured with fixed angle of feather tilt in Figure 11 and Figure 12.

The Computational Fluid Dynamics (CFD) simulation using the finite volume method is carried out through iterative calculations based on the selected turbulence model, depending on the distribution of nodes across the domain or geometry under analysis.

The turbulence model commonly used in numerous previous studies for aircraft wings and wind turbine blade analysis is the k-ω SST (Shear Stress Transport) model. This model enables the observation of wind behavior and its interaction with solid surfaces of interest, as described below.

The values of *k* and *ω* are derived directly from the transport differential equations for turbulent kinetic energy (*k*) and the specific dissipation rate (*ω*). This model represents a combination of Equations (1)–(8), as also referenced in previous works [26,27,28,29], and incorporates the blending function *F*1, as well as turbulence production due to buoyancy effects *P_kb_* y *P_wb_* and the k-w BS model [27].

*Wilcox Model:*

(1) $$\frac{\partial (\rho k)}{\partial t}+\frac{\partial }{\partial x_{j}}\left(\rho U_{j}k\right)=\frac{\partial }{\partial x_{j}}\left(\left(\mu +\frac{\mu_{t}}{\sigma_{k1}}\right)\frac{\partial k}{\partial x_{j}}\;\right)+P_{k}-\beta^{\prime }\rho kw$$

(2) $$\frac{\partial (\rho w)}{\partial t}+\frac{\partial }{\partial x_{j}}\left(\rho U_{j}w\right)=\frac{\partial }{\partial x_{j}}\left(\left(\mu +\frac{\mu_{t}}{\sigma_{k}}\right)\frac{\partial w}{\partial x_{j}}\right)+\alpha_{1}\left(\frac{w}{k}\right)P_{k}-\beta_{1}\rho kw^{2}$$

*k–ε Model:*

(3) $$\frac{\partial (\rho k)}{\partial t}+\frac{\partial }{\partial x_{j}}\left(\rho U_{j}k\right)=\frac{\partial }{\partial x_{j}}\left(\left(\mu +\frac{\mu_{t}}{\sigma_{k2}}\right)\frac{\partial k}{\partial x_{j}}\;\right)+P_{k}-\beta^{\prime }\rho kw$$

(4) $$\frac{\partial (\rho w)}{\partial t}+\frac{\partial }{\partial x_{j}}\left(\rho U_{j}w\right)=\frac{\partial }{\partial x_{j}}\left(\left(\mu +\frac{\mu_{t}}{\sigma_{k}}\right)\frac{\partial w}{\partial x_{j}}\;\right)+2\rho \left(\frac{1}{\sigma_{w2}w}\right)\frac{\partial k}{\partial x_{j}}\frac{\partial w}{\partial x_{j}}+\alpha_{2}\left(\frac{w}{k}\right)P_{k}-\beta_{2}\rho kw^{2}$$

*BSL* *Model*:

(5) $$\frac{\partial (\rho k)}{\partial t}+\frac{\partial }{\partial x_{j}}\left(\rho U_{j}k\right)=\frac{\partial }{\partial x_{j}}\left(\left(\mu +\frac{\mu_{t}}{\sigma_{k3}}\right)\frac{\partial k}{\partial x_{j}}\;\right)+P_{k}-\beta^{\prime }\rho kw+P_{kb}$$

(6) $$\frac{\partial (\rho w)}{\partial t}+\frac{\partial }{\partial x_{j}}\left(\rho U_{j}w\right)=\frac{\partial }{\partial x_{j}}\left(\left(\mu +\frac{\mu_{t}}{\sigma_{w3}}\right)\frac{\partial w}{\partial x_{j}}\;\right)+\left(1-F1\right)2\rho \left(\frac{1}{\sigma_{w2\;}w}\right)\frac{\partial k}{\partial x_{j}}\frac{\partial w}{\partial x_{j}}+{\;\alpha }_{3}\frac{w}{k}P_{k}-\beta_{3}\rho w^{2}+P_{wb}$$

(7) $$P_{k}=\mu_{t}\left(\frac{\partial U_{i}}{\partial x_{j}}+\frac{\partial U_{j}}{\partial x_{i}}\right)\frac{\partial U_{i}}{\partial x_{j}}-\frac{2}{3}\left(\frac{\partial U_{k}}{\partial x_{k}}\right)\left(3\mu_{t}\frac{\partial U_{k}}{\partial x_{k}}+\rho k\right)$$

(8) $$P_{wb}=\frac{w}{k}({(\alpha +1)C}_{3}\max\left(0,\;P_{kb}\right)\sin\phi -P_{kb})$$

where the dynamic viscosity of the fluid is denoted by *μ*, the turbulent viscosity by *μ_t_*, the fluid density by ρ , the turbulent kinetic energy by *k*, the specific dissipation rate by *w*, y and *ϕ* represents the angle between the velocity vector and the gravity vector [27]. Finally, *F1* is a blending function that equals one near the surface and gradually decreases to zero outside the boundary layer.

*β*’ = 0.09, *α*_1_ = 5/9, *β*_1_ = 0.075, *σ*_*K*1_ = 2, *σ*_*W*1_ = 2, *α*_2_ = 0.44, *β*_2_ = 0.082, *σ*_*K*2_ = 1, *σ*_*W*2_ = 1/0.856. (9)

Using the CFX solver in ANSYS^®^, the constant values are determined through a linear combination of the three turbulence models, resulting in the *k–ω* BSL (baseline) model, Equation (5). Similarly, the *k–ω* BSL model combines the advantages of both the Wilcox *k–ω* model and the *k–ε* model; however, it has limitations in accurately predicting the onset and extent of flow separation over smooth surfaces [27].

**Figure 18:**
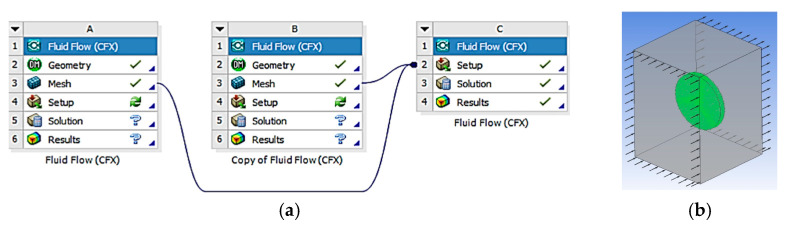
(**a**) CFD block diagram; (**b**) region and domain configuration.

Figure 18 shows the block diagram of the 3D CFD simulation using the CFX solver, configured to simulate an HAWT (Horizontal Axis Wind Turbine). The simulation parameters are detailed in Table 1.

Mesh independence was analyzed through several preliminary simulations using 3D element sizes of 1 mm, 0.8 mm, and 0.5 mm along the edges. The TKE (Turbulent Kinetic Energy) values showed greater stability from 0.8 mm onwards. Therefore, an element size of 1 mm was selected for the blade surface to ensure a balance between computational cost and the accuracy of the CFD simulation results. A mesh refinement was applied, allowing smaller elements to be concentrated in areas of interest, such as near the blade surfaces.

**Figure 19:**
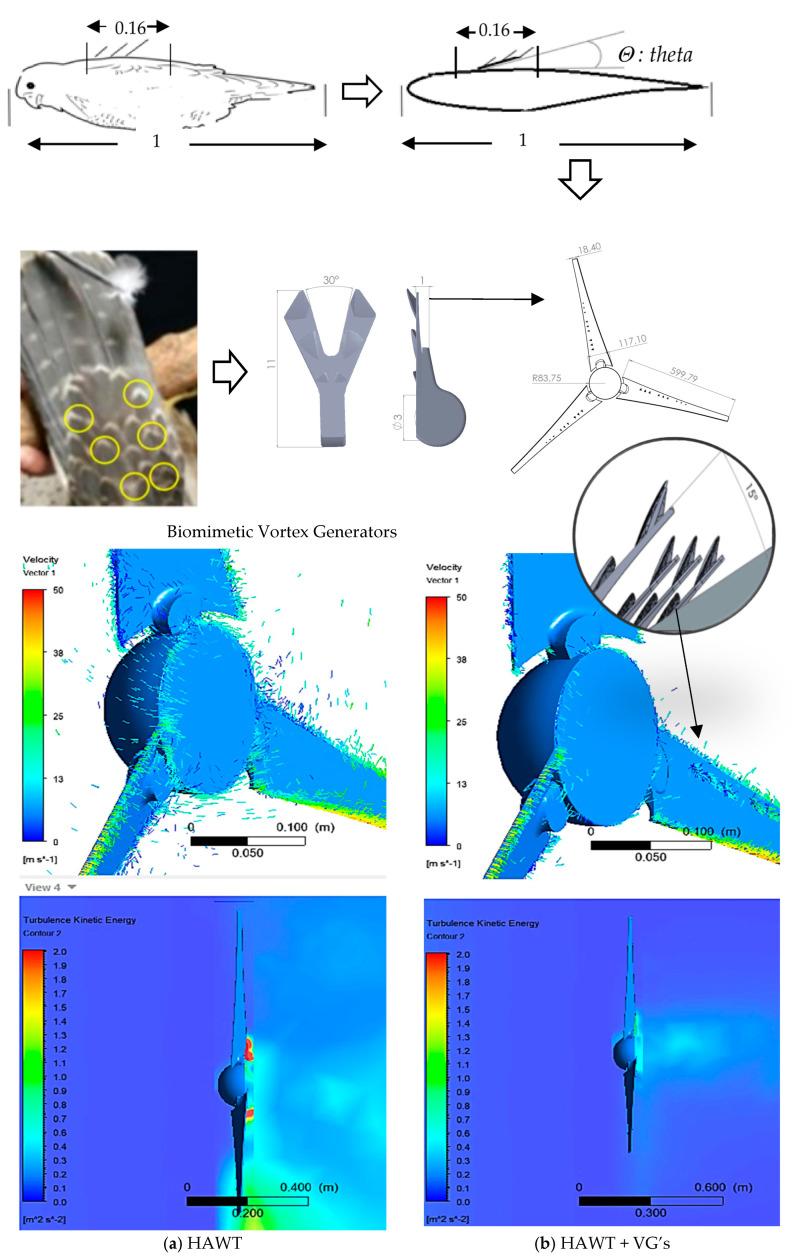
CFD simulation transient—3D.

After studying the biomimetic vortex generators inspired by the dorsal feathers of the peregrine falcon [13], and based on previous analyses of the bird using photographic evidence, the unique behavior of these specialized feathers at high diving speeds was identified. Consequently, the vortex generators were positioned linearly along the suction side of the CAD model of the HAWT-type turbine blades, (Figure 19), at various inclination angles, replicating the natural orientation of the falcon’s feathers along the blade’s airfoil.

## 4. Discussions

Passive flow control mechanisms rely on modifying the surface geometry of structures exposed to the flow, without requiring additional energy input. A notable example is the study by Shen [20], where fish scale-inspired structures were applied to axial compressors to control secondary flow effects. These biomimetic surfaces generated upward vortices that energized low-speed fluid near the wall, suppressed corner vortices, and reduced total pressure loss by up to 5.69%. The simplicity, robustness, and energy-free operation of such designs make them highly suitable for industrial systems operating under harsh or maintenance-limited conditions.

In contrast, active flow control methods require external energy input, offering the ability to dynamically adjust the flow. In our current study, inspired by the oscillating feathers of the peregrine falcon, an experimental prototype with active vortex generators is investigated. These generators demonstrate a clear impact on the spectral distribution of the wake velocity and on the attenuation of structural vibrations. At wind speeds between 10 and 20 m/s, the oscillating feathers led to reduced spectral variance and improved vibration stability. These findings complement earlier work by [28], extending the analysis from aerodynamic performance to include wake dynamics and structural responses.

Both passive and active mechanisms offer advantages depending on the application context. Passive strategies—like the riblet and scaly structures proposed by Shen [20] and self-deployable surfaces studied by Wang [29]—excel in environments where simplicity and durability are critical. Meanwhile, active systems—such as those presented in our falcon-inspired study or in the work by Rosti [30]—offer adaptability through real-time interaction with unsteady flows. Future developments are expected to explore hybrid bioinspired solutions that integrate passive resilience with active responsiveness to optimize aerodynamic control in systems like wind turbines, aircraft, and drones.

The 3D acceleration measurements reveal the vibration levels experienced by the prototype under varying wind tunnel velocities. These acceleration values remain below 1 g, and therefore, the mean value was subtracted to highlight the fluctuation patterns. The difference between peak acceleration values along the X and Z axes remains within the range of −0.06 g to 0.06 g, indicating that the developed vibration measurement system can capture the prototype’s dynamic response with sufficient sensitivity.

The RMS (Root Mean Square) values derived from wind velocity signals obtained using the hot-wire sensor show a direct correlation with increasing wind tunnel speeds. However, as the feather inclination angle increases, a noticeable reduction in RMS values is observed. When the mean value is removed from the velocity signal, RMS values with higher variance are obtained, reflecting the impact of feather movement on wind wake.

FFT spectral analysis of the wind velocity signals measured with the digital hot-wire sensor demonstrates a uniform increase in spectral component magnitudes as wind tunnel speed increases for fixed feather angles.

In contrast, when the feather inclination varies dynamically at a constant frequency, the resulting spectra exhibit higher-magnitude components and distinct frequency bands or peaks associated with the feathers’ oscillatory motion. This indicates that changes in the wind wake velocity due to feather movement are more evident in the frequency domain under oscillatory conditions.

Further analysis of the frequency domain reveals the presence of both low-frequency and high-frequency components, each corresponding to different flow phenomena. The low-frequency components (typically below 2 Hz) are primarily associated with large-scale coherent structures in the wake, such as vortex shedding and periodic flow separation, which are influenced by the overall motion of the feathers. The feathers’ oscillations at 1 Hz, selected based on the peak energy in the spectral density, directly interact with these large-scale turbulent structures, modifying their development and spatial coherence.

On the other hand, the high-frequency components (above 2 Hz) are linked to smaller-scale turbulent eddies and local flow disturbances. The introduction of oscillating feathers appears to energize specific high-frequency bands, likely due to the generation of small-scale vortices or instabilities caused by the unsteady feather motion. These interactions enhance the complexity of the turbulence spectrum, suggesting a multi-scale influence of feather dynamics on flow structure. By modifying both the amplitude and frequency distribution of turbulence, the feather motion plays an active role in flow control, potentially mimicking the natural flow manipulation observed in bird flight. This highlights the relevance of integrating bio-inspired dynamic elements into aerodynamic systems, not only for vibration attenuation but also for turbulence management and performance.

The experimental results suggest that bio-inspired vortex generators (VGs) with oscillating feather-like elements exhibit a dynamic interaction with the flow field that is strongly dependent on wind speed and turbulence intensity. At low wind speeds, the feathers produce mild fluctuations that primarily affect the large-scale coherent structures in the wake. This interaction is reflected in the increased energy content of low-frequency spectral components, indicating that the feathers can influence vortex shedding and flow separation at these regimes, potentially delaying stalls and improving lift distribution along the blade surface.

As wind speed increases, the oscillating VGs continue to influence the flow, but their effect becomes more pronounced in the higher-frequency spectral bands. The increased turbulence intensity causes the unsteady motion of the feathers to generate additional small-scale vortical structures, enhancing mixing in the boundary layer. This effect is beneficial in reducing flow separation and improving aerodynamic efficiency, particularly in the mid- to high-angle-of-attack regions. The ability of the bio-inspired devices to adapt their response through oscillation facilitates flow control that is both passive (through their shape) and active (through their movement), mimicking biological mechanisms found in bird flight.

From an applied perspective, these findings highlight the potential of using bio-inspired vortex generators in real-world wind turbine applications. Their effectiveness under varying wind conditions demonstrates versatility and robustness, key qualities for components operating in outdoor, unsteady environments. The integration of actively controlled oscillating elements could further enhance performance, enabling turbines to self-adjust to changes in turbulence levels and wind speed in real time. Future work should focus on long-term durability, control strategies, and scalability of these systems to full-size turbines.

Unlike the previous studies published in *Energies* [31], by the same group of authors, which focused primarily on the aerodynamic evaluation of bio-inspired vortex generators (VGs), whether fixed or oscillatory, the present work introduces a completely new approach oriented toward the structural and spectral analysis of the system. Rather than evaluating only the flow behavior over the blade, this study focuses on the effects of active vortex generators (with oscillating feathers inspired by the peregrine falcon) on the structural vibrations of the blade and on the spectral distribution of the wake velocity. In this new work, a deeper spectral analysis is conducted on the dynamic behavior of the falcon-inspired prototype with oscillating feathers under different wind speeds, which was not addressed in the previous studies.

## 5. Conclusions

This study demonstrates the potential of bio-inspired, actively oscillating vortex generators (VGs) to modulate wake dynamics and reduce structural vibrations in aerodynamic systems, particularly wind turbine blades. Through a combination of experimental measurements and CFD simulations, we observed that feather-inspired oscillating devices can influence both low- and high-frequency components of turbulence, directly affecting wake stability and flow coherence.

The spectral analysis (FFT) of the velocity signals revealed that dynamic feather oscillations lead to the emergence of well-defined spectral bands, particularly at lower frequencies (around 1 Hz), corresponding to the selected oscillation frequency. These low-frequency components are associated with large-scale wake structures, such as vortex shedding, while the presence of additional high-frequency components suggests an energizing effect on smaller turbulent eddies. This dual-scale interaction mimics the natural flow control mechanisms observed in bird flight, supporting the relevance of dynamic bio-inspired strategies in aerodynamics.

CFD results further confirmed that turbines equipped with vortex generators exhibit reduced kinetic energy regions in the Y-Z wake contour, in comparison to standard blade configurations. This indicates a suppression of turbulence intensity and improved energy recovery potential downstream. The agreement between time-domain experimental measurements and numerical predictions strengthens the credibility of the proposed mechanism.

Given the promising results, several concrete steps are proposed for integrating these findings into practical wind turbine design as follows: Hybrid Passive–Active Systems: The oscillating VGs demonstrated in this study can be further developed into hybrid systems that combine passive shape optimization with active, sensor-driven control. These systems would be capable of adjusting oscillation frequency and amplitude in real time based on environmental conditions (e.g., wind speed, turbulence intensity). Adaptive Flow Control: The feather-inspired actuation mechanism can be scaled and adapted to blade sections most susceptible to stall or flow separation. Real-time flow sensing combined with embedded actuation could enable local adjustments to boundary layer behavior, improving lift distribution and delaying stall onset under unsteady flow conditions. Wake Management for Wind Farms: By attenuating turbulence and modifying wake structure, these devices could reduce wake interactions between turbines in wind farms. Optimizing wake recovery has the potential to increase overall farm efficiency and reduce fatigue loads on downstream turbines.

## Figures and Tables

**Table 1:** CFD simulation parameters.

Simulation Parameters	
Simulation Type	Transient
Turbulence Model	kw-SST
Total Simulation Time	5 s
Time Step	0.04 s
Iterations	800
Fluid	Air at 25 °C
Wind	20 m/s
Pressure	1 atm
Turbulence Level	5%
Subdomain Rotational Speed	1 rev/s

## Data Availability

Data available on request due to restrictions (e.g., privacy, legal or ethical reasons): The data presented in this study are available on request from the corresponding author due to ethical restrictions.

## References

[1] Deshmukh S., Bhattacharya S., Jain A., Paul A.R. (2019). Wind turbine noise and its mitigation techniques: A review. Energy Procedia.

[2] Bodling A., Sharma A. (2019). Numerical investigation of noise reduction mechanisms in a bio-inspired airfoil. J. Sound Vib..

[3] Zhou T., Guo J. (2022). Underwater noise reduction of offshore wind turbine using compact circular liner. Appl. Energy.

[4] Bodling A., Sharma A. Noise Reduction Mechanisms due to Bio-Inspired Airfoil Designs. Proceedings of the 17th International Symposium on Transport Phenomena and Dynamics of Rotating Machinery (ISROMAC2017).

[5] Mystkowski A. (2013). Piezo-stack vortex generators for boundary layer control of a delta wing micro-aerial vehicle. Mech. Syst. Signal Process..

[6] Mereu R., Passoni S., Inzoli F. (2019). Scale-resolving CFD modeling of a thick wind turbine airfoil with application of vortex generators: Validation and sensitivity analyses. Energy.

[7] Zhang X., Xiao H., Gomez T., Coutier-Delgosha O. (2020). Evaluation of ensemble methods for quantifying uncertainties in steady-state CFD applications with small ensemble sizes. Comput. Fluids.

[8] Shirzadi M., Tominaga Y. (2022). CFD evaluation of mean and turbulent wind characteristics around a high-rise building affected by its surroundings. Build. Environ..

[9] Zeng F., Lei C., Liu J., Niu J., Gao N. (2020). CFD simulation of the drag effect of urban trees: Source term modification method revisited at the tree scale. Sustain. Cities Soc..

[10] Whitehouse G.R., Boschitsch A.H. (2021). Investigation of grid-based vorticity-velocity large eddy simulation off-body solvers for application to overset CFD. Comput. Fluids.

[11] Ponitz B., Triep M., Brücker C. (2014). Aerodynamics of the Cupped Wings During Peregrine Falcon’s Diving Flight. Open J. Fluid Dyn..

[12] Ponitz B., Schmitz A., Fischer D., Bleckmann H., Brücker C., Aegerter C.M. (2014). Diving-Flight Aerodynamics of a Peregrine Falcon (*Falco peregrinus*). PLoS ONE.

[13] Gowree E.R., Jagadeesh C., Talboys E., Lagemann C., Brücker C. (2018). Vortices enable the complex aerobatics of peregrine falcons. Commun. Biol..

[14] Shi S.-X., Liu Y.-Z., Chen J.-M. (2012). An Experimental Study of Flow Around a Bio-Inspired Airfoil at Reynolds Number 2.0 × 103. J. Hydrodyn..

[15] Srinivas K.S., Datta A., Bhattacharyya A., Kumar S. (2018). Free-stream characteristics of bio-inspired marine rudders with different leading-edge configurations. Ocean Eng..

[16] Post M.L., Decker R., Sapell A.R., Hart J.S. (2018). Effect of bio-inspired sinusoidal leading-edges on wings. Aerosp. Sci. Technol..

[17] Hassanalian M., Throneberry G., Abdelkefi A. (2017). Wing shape and dynamic twist design of bio-inspired nano air vehicles for forward flight purposes. Aerosp. Sci. Technol..

[18] Xia H., Sun Q., Liu Y. (2022). Energy Absorption Characteristics of Bio-Inspired Honeycomb Column Thin-Walled Structure under Low Strain Rate Uniaxial Compression Loading. Energies.

[19] Zhang Y., Wang W., Ding X., Sun L., Qian Z., Jiang H., Song Y., Ding R. (2025). The Effects of Turbulent Biological Tissue on Adjustable Anomalous Vortex Laser Beam. Biomimetics.

[20] Shen J.-L., Yang H.-C., Yeh S.-I. (2025). Fish Scale-Inspired Flow Control for Corner Vortex Suppression in Compressor Cascades. Biomimetics.

[21] Dvorak P. Conformal Vortex Generator and Elastomer Tab Let NREL Test Turbine Produce 22% More Power. https://www.windpowerengineering.com/conformal-vortex-generator-elastomer-tab-let-nrel-test-turbine-produce-22-power/.

[22] Lattin C.R., Emerson M.A., Gallezot J.-D., Mulnix T., Brown J.E., Carson R.E. (2018). A 3D-printed modular device for imaging the brain of small birds. J. Neurosci. Methods.

[23] Heydari M., Sadat-Hosseini H. (2020). Analysis of propeller wake field and vortical structures using k−ω SST Method. Ocean Eng..

[24] Van Sluis M., Nasrollahi S., Rao A.G., Eitelberg G. (2022). Experimental and Numerical Analyses of a Novel Wing-In-Ground Vehicle. Energies.

[25] Ung S.-K., Chong W.-T., Mat S., Ng J.-H., Kok Y.-H., Wong K.-H. (2022). Investigation into the Aerodynamic Performance of a Vertical Axis Wind Turbine with Endplate Design. Energies.

[26] Aziz S., Khan A., Shah I., Khan T.A., Ali Y., Sohail M.U., Rashid B., Jung D.W. (2022). Computational Fluid Dynamics and Experimental Analysis of a Wind Turbine Blade’s Frontal Section with and without Arrays of Dimpled Structures. Energies.

[27] ANSYS, Inc. (2011). ANSYS CFX-Solver Theory Guide.

[28] Parra H.G., Ceron H.D., Gomez W., Gaona E.E. (2023). Experimental Analysis of Oscillatory Vortex Generators in Wind Turbine Blade. Energies.

[29] Gan W., Wang Y., Wang H., Zhuang J. (2024). Aerodynamic Investigation on a Coaxial-Rotors Unmanned Aerial Vehicle of Bionic Chinese Parasol Seed. Biomimetics.

[30] Rosti M.E., Omidyeganeh M., Pinelli A. (2018). Passive control of the flow around unsteady aerofoils using a self-activated deployable flap. J. Turbul..

[31] Parra H.G., Ceron H.D., Gomez W., Gaona E.E. (2023). Experimental Analysis of Bio-Inspired Vortex Generators on a Blade with S822 Airfoil. Energies.

