# Correction: Parra et al. Experimental and Spectral Analysis of the Wake Velocity Effect in a 3D Falcon Prototype with Oscillating Feathers and Its Application in HAWT with Biomimetic Vortex Generators Using CFD. *Biomimetics* 2025, *10*, 622

**DOI:** 10.3390/biomimetics11030158

**Published:** 2026-02-27

**Authors:** Hector G. Parra, Javier A. Guacaneme, Elvis E. Gaona, Hernán Dario Cerón-Muñoz

**Affiliations:** 1 Engineering Faculty Cajicá, Universidad Militar Nueva Granada, Bogotá 111321, Colombia; 2 Facultad de Ingeniería, Universidad Distrital Francisco José de Caldas, Bogotá 110231, Colombia; jguacaneme@udistrital.edu.co (J.A.G.); egaona@udistrital.edu.co (E.E.G.); 3 Escola de Engenharia de São Carlos, Universidade de São Paulo, São Carlos 13566-590, Brazil; hernan@sc.usp.br

Hernán Dario Cerón-Muñoz was not included as an author in the original publication [1]. The corrected Author Contributions statement appears here. 

**Author Contributions:** Investigation: H.G.P., J.A.G., H.D.C.-M. and E.E.G.; writing—original draft preparation: H.G.P.; supervision, E.E.G. All authors have read and agreed to the published version of the manuscript.

Affiliation 3 was also newly added.

The authors state that the scientific conclusions are unaffected. This correction was approved by the Academic Editors. The original publication has also been updated.
